# What Is Your Diagnosis? Leukocytosis With Abnormal WDF and WNR Scattergrams From the Sysmex XN‐1000V in a Dog

**DOI:** 10.1111/vcp.70092

**Published:** 2026-01-18

**Authors:** Javier Martínez‐Caro, Marta Lemos, Josep Pastor

**Affiliations:** ^1^ Servei d'Hematologia Clínica Veterinària, Departament de Medicina i Cirurgia Animals, Facultat de Veterinària Universitat Autònoma de Barcelona Bellaterra Spain

**Keywords:** canine, hematology, mast cell, mastocytemia, WDF scattergram, WNR scattergram

## 1 Case Presentation

A 12‐year‐old neutered female Cocker Spaniel dog was presented for consultation due to the presence of a non‐pigmented mass on the right mandible. The dog had a previous history of oral melanoma excision. Surgical removal of the new mass was recommended. During preoperative staging, computed tomography revealed a hepatic mass (8 × 12 cm), mandibular and retropharyngeal lymphadenopathy, a gastric lesion consistent with a polyp, and multiple thoracic intervertebral disc protrusions. Cytology of the lymph nodes was consistent with a reactive process.

A liver lobectomy was performed, and histopathological analysis confirmed a grade I hepatocellular carcinoma with complete surgical margins and no evidence of lymphatic or vascular invasion. Subsequently, a right mandibulectomy was also performed, and the mass was confirmed to be an oral melanoma on histopathology.

One month later, the dog was presented due to abdominal distension. On physical examination, the dog was alert but apathetic, with abdominal distension, pale mucous membranes, tachycardia, and normothermia. An abdominal ultrasonography was performed, showing marked peritoneal effusion; a heterogeneous liver with irregular margins in one of the lobes; generalized lymphadenopathy; and a heterogeneous spleen with hypoechoic lesions distorting its margins.

A complete blood count (CBC) was performed using a ProCyte Dx (IDEXX, Westbrook, USA) (Table S1 and Figure S1) and repeated in a Sysmex XN‐1000V (Sysmex Corporation, Norderstedt, Germany) (Table 1 and Figure 1).

**TABLE 1:** Hematology results for the EDTA‐blood specimen of the dog from this report, performed on the Sysmex XN‐1000V.

Variable	Sysmex XN results	Sysmex XN results (manual analysis [extended])	Manual results	Reference interval
RBC (×10^12^/L)	**2.28**			5.5–8.5
HCT (%)	**18.4**		**18** (PCV)	37–55
HGB (g/dL)	***5.8***			12–18
MCV (fL)	**80.7**			62–77
MCH (pg)	25.4			21.5–26.5
MCHC (g/dL)	**31.5**			33–37
RET (×10^9^/L)	57.7			0–60
RET‐He (pg)	**25.8**			> 20.9
nRBC (cells/100WBC)	**3.4**		**5**	≤ 1
WBC (×10^9^/L)	**23.27**	**24.35**	**(23.27)**	6–17
Neutrophils (×10^9^/L)	4.59	5.23	5.82 (25%)	3–11.5
Lymphocytes (×10^9^/L)	**8.88**	2.22	**0.47** (2%)	1–4.8
Monocytes (×10^9^/L)	**1.77**	**2**	**1.4** (6%)	0.15–1.35
Eosinophils (×10^9^/L)	0.94	0.14	0 (0%)	0.1–1.5
Basophils (×10^9^/L)	**7.09**	—	0 (0%)	0–0.2
Mast cells (×10^9^/L)	—	**14.74**	**15.58** (67%)	0
PLT‐I (×10^9^/L)	**52**			200–500
PLT‐O (×10^9^/L)	**78**			200–500
Estimated PLT (×10^9^/L)	—	—	195–260	200–500

*Note:* Left column, automated results; middle column, results after manual regating (using manual analysis [extended]); right column, manual/blood smear results. The WBC differential count was performed by J.M.C. based on a 200‐cell count. The PCV was obtained by standard microhematocrit capillary centrifugation. Bolded values are outside the reference interval.

Abbreviations: HCT, hematocrit; HGB, hemoglobin; MCH, mean corpuscular hemoglobin; MCHC, mean corpuscular hemoglobin concentration; MCV, mean cell volume; nRBC, nucleated RBC; PCV, packed cell volume; PLT‐I, platelets impedance; PLT‐O, platelets optical; RBC, red blood cells; RET, reticulocytes; RET‐HE, reticulocyte hemoglobin equivalent; WBC, white blood cells.

**FIGURE 1:**
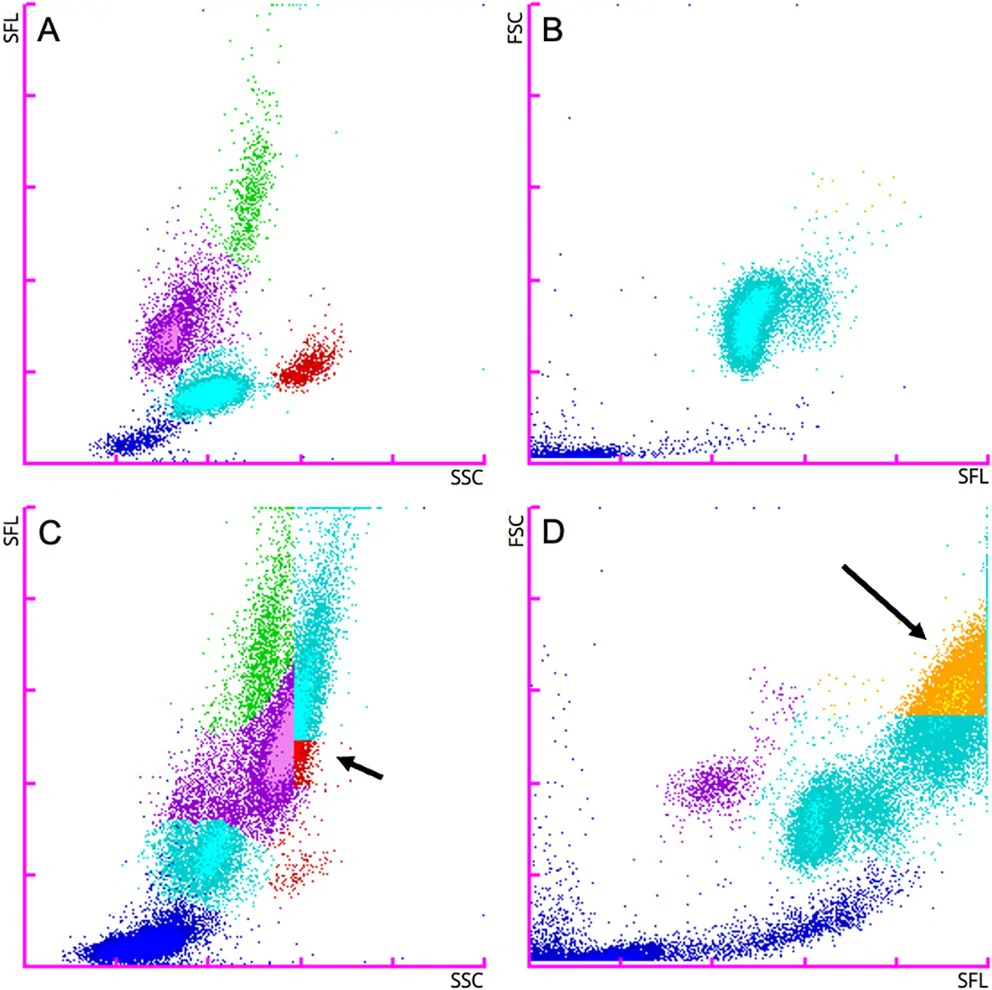
Canine peripheral blood scattergrams from the Sysmex XN‐1000V. (A) and (B) show an example of a healthy dog. (C) and (D) show the blood of the dog from this case report. A and C show WDF scattergrams, and B and D show WNR scattergrams. The short arrow on the WDF scattergram indicates an abnormal cell population characterized by high fluorescence and complexity (high SFL and SSC, respectively). The long arrow on the WNR scattergram indicates a population of larger‐sized cells (higher FSC), indicating lysis‐resistant properties. FSC, forward scattered light intensity; SFL, side fluorescence light intensity; SSC, side scattered light intensity. Particle representation: in WDF channel (A and C), cyan dots (neutrophils), purple dots (lymphocytes), green dots (monocytes), red dots (eosinophils), and dark blue dots (debris); in WNR channel (B and D), cyan dots (white blood cells [except basophils]), purple dots (nucleated red blood cells), yellow dots (basophils—lysis resistant cells), and dark blue dots (debris).

### 1.1 Scattergram Interpretation

Atypical scattergrams due to an abnormal cluster of cells with high fluorescence and complexity (WDF scattergram) which are lysis resistant (WNR scattergram). These properties suggest the presence of granular cells with properties compatible with mast cells. A neoplastic mastocytemia is highly suspected.

Blood smears were performed, air dried, and stained on a Hematek 2000 (Siemens Healthcare Diagnostics, Deerfield, IL, USA) using a Wright‐Giemsa stain (Hematek Stain Pak—Wright‐Giemsa stain, Siemens Healthcare Diagnostics, Deerfield, IL, USA). The blood smear review showed the presence of high numbers of well differentiated mast cells (Table 1 and Figure 2), and the leukocytosis was attributed to the severe mastocytemia. The overall hematological interpretation was marked macrocytic, hypochromic, non‐regenerative anemia with inappropriate rubricytosis, and leukocytosis due to marked mastocytemia, with mild monocytosis and moderate lymphopenia.

**FIGURE 2:**
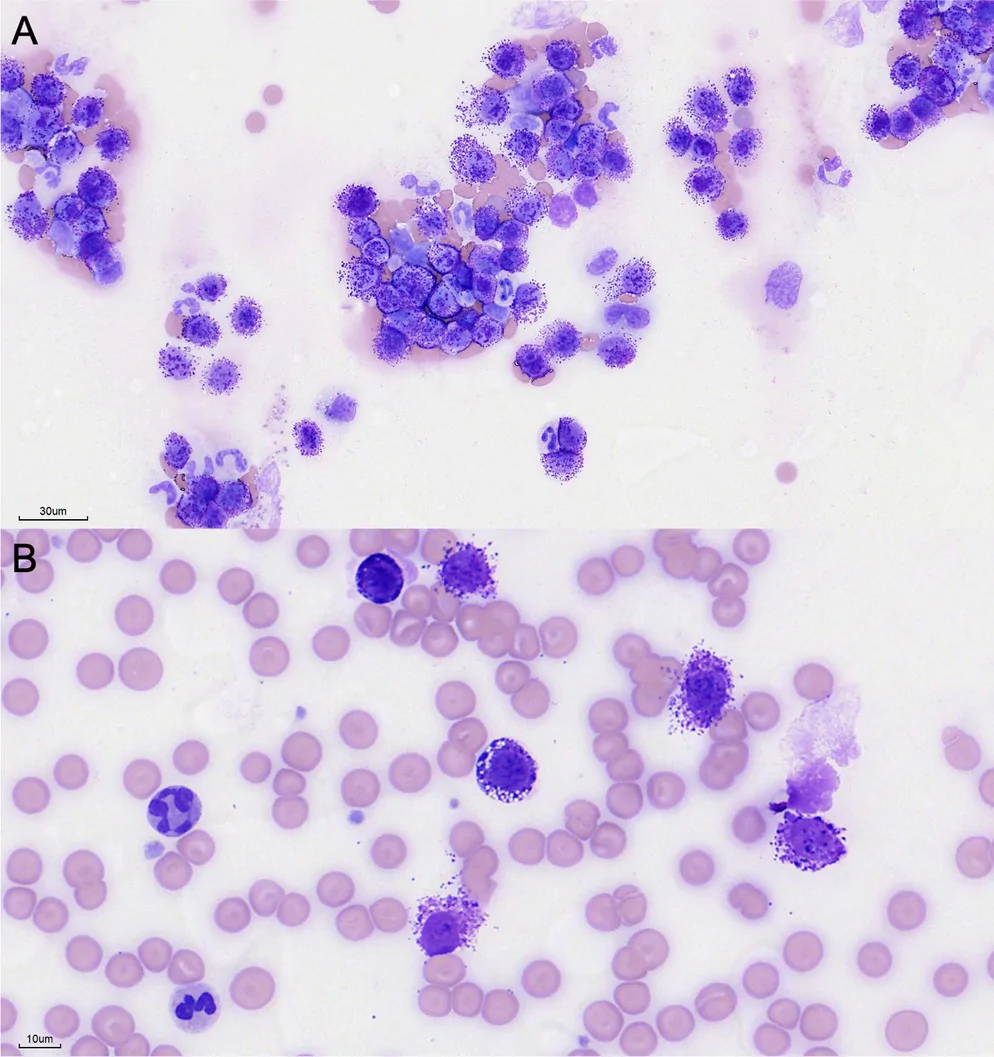
Peripheral blood smear micrographs from the dog of this report (A, B). Note the high numbers of mast cells (67%), characterized by their abundant metachromatic cytoplasmic granules. Wright‐Giemsa stain, images acquired using a digital slide scanner (Motic EasyScan One, MoticEurope SLU, Barcelona, Spain).

## 2 Additional Results

Manual WDF regating was performed in the manual analysis (extended) mode (Table 1 and Figure 3), showing a similar proportion of mast cells as manually calculated. Unlike the manual analysis mode that just allows modifying the predefined gates, the manual analysis (extended) mode allows creating the desired number of gates. Sero‐hemorrhagic fluid from the abdominal effusion was analyzed, with a total nucleated cell count of 1.14 × 10^9^/L, a RBC count of 0.02 × 10^12^/L, and total proteins (refractometry) of 5 g/dL, consistent with a protein‐rich transudate. Cytological evaluation (Figure S2) showed mast cells (43%), non‐degenerated neutrophils (32.5%), small lymphocytes (12%), macrophages (9.5%), and eosinophils (3%). The mast cells showed minimal granulation on aqueous Romanowsky staining and were moderately to well granulated with Wright‐Giemsa stain. They showed moderate cellular atypia, including moderate anisocytosis and anisokaryosis, and increased nuclear‐to‐cytoplasmic ratio. The interpretation was neoplastic effusion due to a mast cell tumor. Ultrasound‐guided fine needle aspiration of a hepatic lymph node was performed. Cytological examination revealed a moderately differentiated mast cell tumor, with scant lymphoid tissue and eosinophilic inflammation. The overall interpretation was neoplastic mastocytemia and effusion, possibly due to a visceral mast cell tumor. The owners declined further investigations or treatment, and the dog was euthanized. Necropsy was not performed.

**FIGURE 3:**
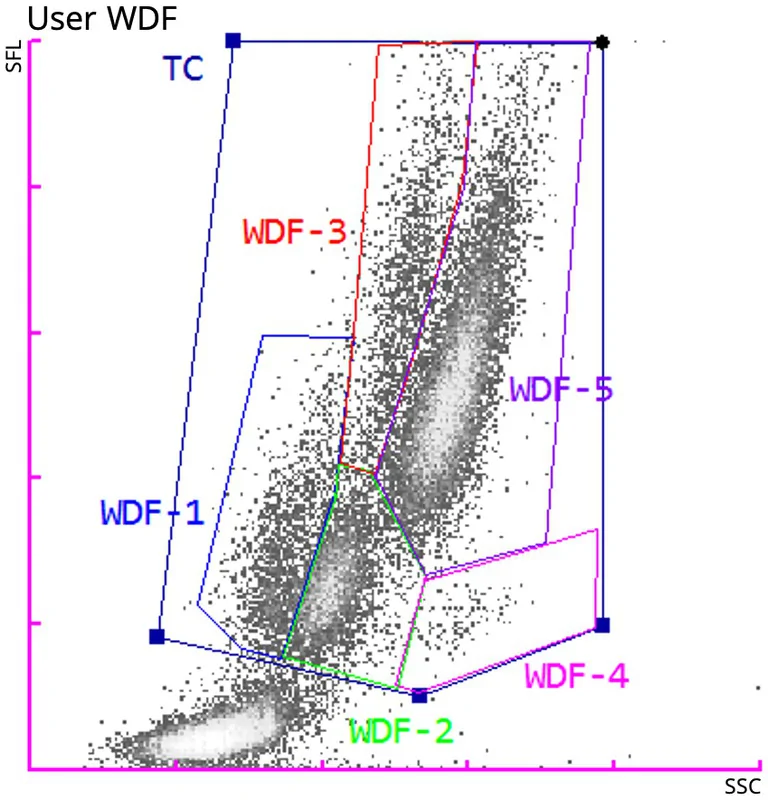
Canine peripheral blood WDF scattergram from the Sysmex XN‐1000V after regating in the manual analysis (extended) mode. Mast cells represent 60.5% of total events. SFL, side fluorescence light intensity; SSC, side scattered light intensity; TC, total cells; WDF‐1, lymphocytes; WDF‐2, neutrophils; WDF‐3, monocytes; WDF‐4, eosinophils; WDF‐5, mast cells.

## 3 Discussion

The Sysmex XN‐1000V is an updated version of the Sysmex XT‐2000*i*V. Several changes have been made to the new analyzer, including the incorporation of a platelet fluorescence channel. The DIFF scattergram in the Sysmex XT‐2000*i*V has been replaced by the WDF scattergram in the Sysmex XN‐1000V. While the WDF scattergram is similar overall, some differences in cell distribution have recently been described, such as the feline basophil distribution [1, 2, 3], which now appears higher on the *y*‐axis (higher fluorescence). The BASO scattergram in the Sysmex XT‐2000*i*V has been replaced by the WNR scattergram in the Sysmex XN‐1000V, which now allows for the nucleated RBC (nRBC) enumeration and then reports a corrected absolute WBC count [4, 5]. This channel was developed to recognize human basophils, but similarly to the previous analyzer, true basophil detection does not happen in dogs [4, 6]. Human basophils and those of certain non‐human species, such as rabbits, horses, or hedgehogs (authors' observation), are known to resist the lytic action of the lysing reagent, enabling their detection and enumeration by the Sysmex XN‐1000V.

The characteristics of feline mastocytemia in the Sysmex XN‐1000V have recently been described [3]. In dogs, mastocytemia has been described in the Sysmex XT‐2000*i*V and the ProCyte Dx [7]. In the latter, the findings of our case were similar to those previously described, and mast cells were positioned between monocytes, neutrophils, and eosinophils (Figure S1). This is the first description of the characteristics of circulating canine mast cells in the Sysmex XN‐1000V, which seem to be mostly similar to those of the previous analyzer. The mast cells exhibited high side scatter values on the WDF scattergram, similar to eosinophils, possibly due to their high cytoplasmic granularity. Their high fluorescence and positioning above the eosinophils could be due to a high nucleic acid content and the presence of cytoplasmic granules. Compared with their position in the DIFF scattergram, mast cells appeared higher on the *y*‐axis (higher fluorescence) in the WDF scattergram. The mast cells of our case showed lysis‐resistant properties in the WNR scattergram, resulting in an artifactual increase in basophil count (pseudobasophilia).

In dogs, mastocytemia may occur in the context of both non‐neoplastic and neoplastic conditions, including mast cell neoplasia, and its severity is not necessarily indicative of the presence of a mast cell tumor [8, 9]. In the present case, a mastocytemia of neoplastic origin was considered the main etiological differential diagnosis due to the markedly elevated number of circulating mast cells, the presence of a transudative effusion rich in mast cells, and cytological findings from the hepatic lymph node. A visceral mast cell tumor was considered the most probable origin, given the absence of cutaneous or subcutaneous nodules and the imaging findings. Mast cell leukemia was considered unlikely due to the lack of additional cytopenias beyond the erythroid lineage. However, it could not be completely ruled out, as no further diagnostic procedures, such as splenic or hepatic cytology, bone marrow evaluation, or necropsy, were performed. A mild monocytosis and moderate lymphopenia were possibly due to glucocorticoid exposure.

In conclusion, to the authors' knowledge, this is the first report of canine mastocytemia on the Sysmex XN‐1000V. These hematologic abnormalities underscore the importance of manual review of scattergrams for accurate validation of analyzer results and emphasize the critical role of blood smear evaluation in the interpretation of atypical findings.

## Conflicts of Interest

The authors declare no conflicts of interest.

## Supporting information


**Table S1:** Hematology results for the EDTA‐blood specimen of the dog from this report performed on the ProCyte Dx. Bolded values are outside the reference interval. Abbreviations: HCT, hematocrit; HGB, hemoglobin; MCH, mean corpuscular hemoglobin; MCHC, mean corpuscular hemoglobin concentration; MCV, mean cell volume; MPV, mean platelet volume; PCT, plateletcrit; PLT, platelets; RBC, red blood cells; RET, reticulocytes; RET‐HE, reticulocyte hemoglobin equivalent; RDW, red cell distribution width; WBC, white blood cells.

**Figure S1:** Canine peripheral blood scattergrams from the WBC channel from the ProCyte Dx. (A) shows an example of a healthy dog. (B) shows the blood from the dog from this report. The arrow indicates an abnormal cell population characterized by high fluorescence and complexity (high SFL and SSC respectively). Abbreviations: SFL, side fluorescence light intensity; SSC, side scattered light intensity. Particle representation: purple (neutrophils), dark blue dots (lymphocytes), red dots (monocytes), green dots (eosinophils), cyan dots (basophils), and orange dots (debris).

**Figure S2:** Abdominal fluid from the dog from this report. Note the high numbers of mast cells (43%), which are poorly granulated with aqueous Romanowsky stain and show moderate numbers of metachromatic granules after restaining with Wright‐Giemsa. (A and B) Aqueous Romanowsky stain; (C and D) Wright‐Giemsa stain; images acquired using a digital slide scanner (Motic EasyScan One, MoticEurope SLU, Barcelona, Spain).
