# Supplementary material for: What Is Your Diagnosis? Leukocytosis With Abnormal WDF and WNR Scattergrams From the Sysmex XN‐1000V in a Dog
Source: Vet Clin Pathol. 2026 Jan 18;55(Suppl 1):S12–5. doi: 10.1111/vcp.70092 (PMC13666547; doi:10.1111/vcp.70092)
Supplement: Supplementary file 1 [file VCP-55-S12-s002.docx]

Table S1

| Variable | ProCyte Dx Results | Reference interval |
| --- | --- | --- |
| RBC (x10^12^/L) | **2.55** | 5.65-8.87 |
| HCT (%) | **18.6** | 37.3-61.7 |
| HGB (g/dL) | **6.3** | 13.1-20.5 |
| MCV (fL) | 72.9 | 61.6-73.5 |
| MCH (pg) | 24.7 | 21.2-25.9 |
| MCHC (g/dL) | 33.9 | 32-37.9 |
| RDW (%) | 20.2 | 13.6-21.7 |
| RET (x10^9^/L) | 64 | 10-110 |
| RET-He (pg) | 26.6 | 22.3-29.6 |
| WBC (x10^9^/L) | **36.14** | 5.05-16.76 |
| Neutrophils (x10^9^/L) | **11.89** | 2.95-11.64 |
| Lymphocytes (x10^9^/L) | **5.70** | 1.05-5.10 |
| Monocytes (x10^9^/L) | **18.01** | 0.16-1.12 |
| Eosinophils (x10^9^/L) | 0.22 | 0.06-1.23 |
| Basophils (x10^9^/L) | **0.32** | 0-0.1 |
| PLT (x10^9^/L) | **89** | 148-484 |
| MPV (fL) | **18.6** | 8.7-13.2 |
| PCT (%) | 0.17 | 0.14-0.46 |
